# Evaluation of Plasma Isoprostane in Patients with Oral Lichen Planus

**Published:** 2016-03

**Authors:** Maryam Amirchaghmaghi, Seyed Isaac Hashemy, Banafsheh Alirezaei, Fereshteh Jahed Keyhani, Sanaz Kargozar, Samaneh Vasigh, Shideh Gharaei, Atessa Pakfetrat

**Affiliations:** 1 Oral and Maxillofacial Diseases Research Center, Dept. of Oral Medicine, School of Dentistry, Mashhad University of Medical Sciences, Mashhad, Iran.; 2 Surgical Oncology Research Center, Imam Reza Hospital, Faculty of Medicine, Mashhad University of MedicalSciences, Mashhad, Iran.; 3 Undergraduate Student, Faculty of Dentistry, Mashhad University of MedicalSciences, Mashhad, Iran.

**Keywords:** Oral Lichen Planus, Isoprostane, Oxidative Stress

## Abstract

**Statement of the Problems:**

Lichen planus is a chronic inﬂammatory disease. Free radicals and reactive oxygen species play important roles in pathogenesis of oral lichen planus (OLP). Isoprostanes show oxidative stress and are formed by free-radical mediated lipid peroxidation of arachidonic acid and cell membrane phospholipids.

**Purpose:**

This study was conducted to evaluate the plasma level of 8-isoprostane in patients suffering from erosive and non-erosive forms of OLP.

**Materials and Method:**

In this case-control study, 31 patients with OLP and 30 control subjects were enrolled. Plasma samples were obtained and the level of 8-isoprostane was measured with Sandwich enzyme-linked immunosorbent assay (ELISA) in both groups. The data were analyzed by using two-sample t-test, chi-square and ANOVA tests.

**Results:**

The results showed significant increase in the plasma level of 8-isoprostane in OLP group compared with the control group. The results of independent t-test revealed no significant correlation between the plasma level of isoprostane and sex, smoking, or previous treatment.

**Conclusion:**

Based on the findings of this study, oxidative stress was increased in patients with OLP, reflected by higher concentrations of 8-isoprostane in plasma.

## Introduction

Lichen planus is a chronic inﬂammatory disease[1-2] and common dermatological condition that affects the oral cavity and skin with a prevalence of 1-2% in the general population.[3] Oral lichen planus (OLP) affects the oral mucosa of white females over 40 years old more than others.[4] It has various forms of clinical manifestations such as keratotic, atrophic, bullous, and erosive (ulcerative) lesions that may affect various sites of oral cavity.[5-6]

Although the etiology and pathogenesis of OLP is not well-determined, genetic predisposition, stresses, as well as some of viral and bacterial agents are various factors that may act as risk factors for this disease.[7-10]

The etiology of OLP has become more evident during the recent years and the immune system is proposed to have a primary role in the development of this disease. This theory is supported by the histopathologic characteristics of a subepithelial band-formed infiltrate dominated by T-lymphocytes and macrophages.[5]

On the other hand, it is hypothesized that free radicals and reactive oxygen species (ROS) also have strong relationship with OLP pathogenesis and carcinogenesis.[11] It should be considered that in the histopathologic evaluation of lichen planus, basal cell degeneration, infiltration of inflammatory cells (like T-lymphocytes), and destruction of keratinocytes are observed.[12] Free radicals and the other active oxygen compounds intensify the inflammatory reactions in the presence of T-lymphocytes and destroy the lipid membrane of keratinocytes.[13-14]

Free radicals are able to produce chemical modiﬁcations and damage the proteins, lipids, and nucleotides. Reactive free radicals may damage cells by starting lipid peroxidation that causes deep changes in the structural integrity and functions of cell membranes.[15] The role of stress oxidative indices has been introduced in many autoimmune and inflammatory diseases such as atopic dermatitis, psoriasis vulgaris, vitiligo, and dermal lichen planus. The effect of stress oxidative indices in the pathophysiologic changes occurred in the basal cell of epidermis and they verified the decreased antioxidants defense and an increased oxidative damage to the lipids, DNA and proteins.[16-17]

F2-isoprostanes are a group of bioactive prostaglandins produced by oxidative catalyzed reaction of arachidonic acid. They are discussed as the reliable marker of lipid peroxidation *in vivo*.[18] Isoprostanes can be released into the blood circulation, body secretions and urine.[19] Elevated levels of plasma and/or urinary 8-isoprostane have been reported in several conditions such as diabetes,[20-21] alcoholic liver diseases, and cardiovascular diseases.[22-23] 8-isoprostane has been proposed as a reliable biomarker of oxidative stress and antioxidant deficiency because of its biochemical stability.[24]

The aim of this study was to evaluate the isoprostane plasma level in patients suffering from erosive and keratotic OLP in comparison with the control healthy group. 

## Materials and Method

In this case-control study, 31 participants with consecutive OLP, including 20 females and 11 males were enrolled. They were selected through simple sampling method from patients who referred to the Department of Oral Medicine, Mashhad Dental School, Iran, from October 2012 to June 2013. The controls consisted of 30 healthy individuals (F=18, M=12) without lichen planus, who lived in the same geographic location. All the participants were informed about the research study and agreed to participate by signing an informed consent form. The study was also approved by the Ethical Committee of Mashhad University of Medical Sciences (code: 89336). A clinical diagnosis of OLP was established when reticular or popular textures were clinically present, and based on the histopathologic appearance of the disease including basal cell degeneration, and infiltration of inflammatory cells such as T-lymphocytes.

The exclusion criteria were any previous treatment for OLP in the preceding two months; presence of lichenoid reaction or dysplasia in histopathologic examination of the lesions; presence of any factors which could alter the equilibrium of production and elimination of free radicals (such as cigarette smoking, and alcohol consumption) ;the use of antioxidant supplements (vitamin E and vitamin C), steroids and NSAIDS; and finally any systemic disease such as malignancies or inflammatory diseases which could influence the immune system. After taking an accurate medical history, completing a preliminary checklist, and taking biopsies, 5 ml venous blood samples were taken from each subject on the same day and before starting the treatment. Plasma was separated and samples were stored at -80 °C in the presence of 0.005% butylated hydroxytoluene (BHT) until measurement.

The plasma 8-isoprostane level was measured by using 8-Isoprostane EIA kit (Item No. 516351; Cayman Chemical Co., USA) according to the manufacturer’s instructions and based on a competitive method in which isoprostane was measured by Sandwich ELISA. Briefly, this assay is based on the competition between 8-isoprostane in the sample and a constant concentration 8-isoprostane-acetylcholinesterase (AChE) conjugate (8-isoprostane tracer) for a limited number of 8-isoprostane- specific rabbit antiserum binding sites. The amount of 8-isoprostane tracer that bound to the rabbit antiserum was inversely proportional to the concentration of 8-isoprostane in our samples. All other chemicals were purchased from Sigma-Aldrich, Germany. 

The collected data was analyzed by using SPSS software, version 11.5 (Chicago; IL). Two-sample t-test, chi-square, and ANOVA were used as appropriated. P-value<0.05 was deliberated as the statistical significance in this study. 

## Results

In this study, OLP was classified as non-erosive (reticular, popular, plaque-like) and erosive (erosive, atrophic, erosive-atrophic) forms. Accordingly, a total of 31 patients, comprising 22 cases of erosive-atrophic and 9 cases of keratotic OLP with the age range of 30-79 (46.48±11.08) were included. Also, 30 healthy subjects free of oral mucosal diseases were recruited as matched controls. The two study groups were not significantly different in terms of age and sex distribution (*p*> 0.05).

The achieved results showed a significant increase in the plasma level of 8-isoprostane in OLP group compared with the control group (Table 1).

**Table 1:** Plasma level of 8-isoprostane in the study groups (*p*< 0.001)

	**Mean±SD**	** P value *versus *control **	** P value *versus* other forms of OLP **
Healthy Subjects (n=30)	68.98±18.23		
OLP (n=31)	124.19±28.67	0<0.001	
E-OLP	133.29±26.08	0<0.001	0.349
A-OLP	127.18±29.99	0<0.001	0.831
E,A-OLP	123.74±10.25	0<0.001	0.876
K-OLP	115.38±28.24	0<0.001	0.20

Comparing the erosive and non-erosive OLP revealed the isoprostane plasma level in erosive OLP group was significantly higher than keratotic OLP (*p*< 0.001) (Table 2).

**Table 2:** The mean±SD plasma level of isoprostane in erosive and non-erosive OLP groups

**Group**	**N**	**Mean±SD**	**p-value**
Erosive OLP	18	131.25±30.12	<0.001
Non-erosive OLP	9	115.38±28.24	

## Discussion

The current study measured the 8-isoprostane plasma level in patients with erosive and keratotic OLP, and compared them with the control group. A significant increase was noticed in the 8-isoprostane plasma level in OLP groups compared with the healthy controls. Comparisons showed that the 8-isoprostane level was approximately doubled in subjects with erosive OLP. The highest concentration of 8-isoprostane in plasma was observed in patients with erosive OLP; while, the lesser increase in 8-isoprostane plasma level was detected in patients with keratotic OLP.

Free radicals and ROS play important roles in both pathogenesis of lichen planus and carcinogenesis.[15, 25-26]

Other studies pointed out the impact of stress oxidative indices on the pathophysiologic changes occurred in the basal cells of epidermis and concluded a decreased antioxidant defense and an increased oxidative damage to the lipids, proteins and DNA molecules in patients suffering from OLP.[16, 27]

It was found that ROS produced by keratinocytes, fibroblasts and various inflammatory cells could result in disequilibrium between the pro-oxidants and antioxidants.[28] Reactive oxygen metabolites destruct the cell membranes by lipid peroxidation.[29]

Researchers have proven the role of oxidative stress in the etiopathogenesis of OLP by estimating the levels of oxidative markers like malondialdehyde (MDA) and nitric oxide (NO) in various samples of serum, saliva, and tissue.[30]

Agha Hoseini *et al.* stated that the oxidative stress processes play a role in OLP etiopathogenesis and reported an elevated level of MDA of saliva in OLP as an index of oxidative stress.[15]

In another study conducted on 45 patients with lichen planus and 45 control individuals in Egypt, the serum level of NO increased substantially.[16]

Payeras *et al.* found low levels of uric acid and significantly higher MDA levels in saliva of OLP patients compared with the control group. In serum, however, gamma-glutamyl transferase (GGT) was higher and total antioxidant defense was lower in OLP patients than those in healthy subjects.[31]

Isoprostanes, isomers of PGF2α, emerged as one of the most accurate approaches to assess oxidant injury in vivo.[32]

The discovery of isoprostanes had important implications in medicine. Namely, measurement of F2-isoprostanes provides an important tool to explore the role of oxidative stress in the pathogenesis of human disease; thus, it is the most reliable approach to assess oxidative stress status *in vivo*. In addition, the products of isoprostane pathway have been found to exert potent biological actions and therefore may be pathophysiologic mediators of the disease.[33]

In fact, in the Biomarkers of Oxidative Stress study (BOSS) sponsored by the National Institute of Health (NIH), F2-Isoprostanes were found as the most accurate biomarker to assess *in vivo* oxidant stress status when compared against other well-known biomarkers.[32]

Measurement of F2-isoprostanes has several advantages over other quantitative markers of oxidative stress. F2-Isoprostanes are chemically stable, specific products of peroxidation, formed *in vivo*, present in detectable amounts in all normal tissues and biological fluids, thus allowing definition of a normal range; they are increased substantially in animal models of oxidant injury, and are unaffected by lipid content in the diet, and might provide a sensitive biochemical basis in dose-finding studies with antioxidants.[33]

8-Isoprostane is the best characterized compound belonging to the F2-isoprostanes (a group of stable prostaglandins F2) and is an isomer which is formed by free radical-mediated peroxidation of arachidonic acid independent of the action of cyclooxygenase. For this reason, 8-isoprostane is considered an ideal marker for investigating the pathophysiology of oxidative injury.[33] It is suggested that measurement of F2-isoprostanes may provide a uniquely valuable approach to determine whether improvement of oxidative stress mitigates the manifestations of diseases or not.[33] Further studies are suggested to assess the markers of oxidative stress before and after the treatment in these cases.

## Conclusion

This study showed significant increase in the plasma level of 8-isoprostane in patients with OLP compared with the control group, supporting the hypothesis that oxidative stress may be a potential target for developing new strategies of drug treatments.

Based on the achieved findings, measurement of F2-isoprostanes provides investigators with a unique tool to assess the role of free radicals in the pathogenesis of lichen planus with a degree of reliability that were not possible before. Further studies with larger sample sizes are required to realize the role of 8-Isoprostane and other oxidative stress indices in oral mucosal diseases thoroughly. 
